# P-227. Strategy for the accurate identification of CAUTIs in the ICU at a Tertiary Care Hospital

**DOI:** 10.1093/ofid/ofae631.431

**Published:** 2025-01-29

**Authors:** Christina J Bascara, Fredy Chaparro-Rojas, Sophia Panaccione

**Affiliations:** Lankenau Medical Center - Main Line Health, Yardley, Pennsylvania; Main Line Health, Wynnewood, Pennsylvania; Brown University, Providence, Rhode Island

## Abstract

**Background:**

The rates of CA-UTIs increased significantly after the COVID-19 pandemic. Many of these infections were considered "definitional infections", in which cases, they were considered reportable infections based on NHSN criteria, however, not conclusive by clinical presentation. The IDSA recommends removing and replacing indwelling catheters that have been in place for more than 14 days before obtaining a new urinary sample to evaluate for presence of UTI. This is currently not routinely performed.

**Methods:**

In order to improve the yield of evaluating non-contaminated urine specimens for true infection, we developed a protocol that triggered a catheter exchange if the catheter has remained in place for more than 3 days. The protocol consists of removal and replacement of urinary catheter before processing the urine sample and obtaining a culture.

**Results:**

A significant reduction in the number of UTIs was observed after 12 months of use and surveillance of our strategy despite a stable rate of use of urinary catheters. Our intervention was initiated in February 2023 and only 3 CA-UTIs were identified during this time period. It is important to mention that multiple other strategies were deployed during the COVID-19 pandemic in order to decrease the number of CA-UTIs, including an ED lead initiative to decrease the use of new Foley insertion for patients going to ICU and the use of GCH based perineal care (Pericare). In 2022 a significant decline in CA-UTIs was noted (5 CA-UTIs) and 3 cases in 2023.

Currently, we are processing data and additional variables to help us adjusting our findings for potential confounders.

**Conclusion:**

The implementation of a catheter-exchange program for the accurate identification of catheter associated UTIs in the ICU setting, together with the use of GCH based pericare, are effective strategies to decrease the incidence of Catheter associated UTIs.

**Disclosures:**

**All Authors**: No reported disclosures

